# Axon Regeneration Is Regulated by Ets–C/EBP Transcription Complexes Generated by Activation of the cAMP/Ca^2+^ Signaling Pathways

**DOI:** 10.1371/journal.pgen.1005603

**Published:** 2015-10-20

**Authors:** Chun Li, Naoki Hisamoto, Kunihiro Matsumoto

**Affiliations:** Division of Biological Science, Graduate School of Science, Nagoya University, Chikusa-ku, Nagoya, Japan; University of California, San Francisco, UNITED STATES

## Abstract

The ability of specific neurons to regenerate their axons after injury is governed by cell-intrinsic regeneration pathways. In *Caenorhabditis elegans*, the JNK and p38 MAPK pathways are important for axon regeneration. Axonal injury induces expression of the *svh-2* gene encoding a receptor tyrosine kinase, stimulation of which by the SVH-1 growth factor leads to activation of the JNK pathway. Here, we identify ETS-4 and CEBP-1, related to mammalian Ets and C/EBP, respectively, as transcriptional activators of *svh-2* expression following axon injury. ETS-4 and CEBP-1 function downstream of the cAMP and Ca^2+^–p38 MAPK pathways, respectively. We show that PKA-dependent phosphorylation of ETS-4 promotes its complex formation with CEBP-1. Furthermore, activation of both cAMP and Ca^2+^ signaling is required for activation of *svh-2* expression. Thus, the cAMP/Ca^2+^ signaling pathways cooperatively activate the JNK pathway, which then promotes axon regeneration.

## Author Summary

An axon’s ability to regenerate after injury is governed by cell-intrinsic regeneration pathways. In *C*. *elegans*, the JNK and p38 MAPK pathways play an important role in axon regeneration. The JNK pathway is activated by growth factor SVH-1, which signals through its receptor SVH-2. It is known that expression of the *svh-2* gene is induced in response to axonal injury, however the molecular mechanisms underlying this induction have been unknown. Here, we demonstrate that induction of *svh-2* expression in response to axon injury involves the transcription factors ETS-4 and CEBP-1, which function downstream of the cAMP and Ca^2+^–p38 MAPK pathways, respectively. Our results suggest that these two injury-signaling pathways converge to regulate expression of the *svh-2* gene and thereby promote axon regeneration.

## Introduction

The ability of a neuron to regenerate following injury is dependent on both its intrinsic growth capacity and the extracellular environment. When an axon is injured, intracellular levels of calcium (Ca^2+^) and cyclic adenosine monophosphate (cAMP) increase [1]. The increase in cAMP levels activates protein kinase A (PKA), which in turn activates the axon regeneration-promoting transcription factor CREB. PKA also promotes remodeling of the cytoskeleton, which is necessary for the formation and maintenance of the growth cone, a specialized structure necessary to initiate regeneration. Upon axon severance, regeneration signals are retrogradely transported from sites of damage and imported into the nucleus, where they induce the up-regulation of several transcription factors and drive the increased synthesis of proteins involved in neurite outgrowth [2,3]. Manipulation of these processes can improve the chances for successful axon regeneration. Nonetheless, our understanding of the intrinsic signaling pathways that promote this regenerative ability remains limited.

**Fig 1:**
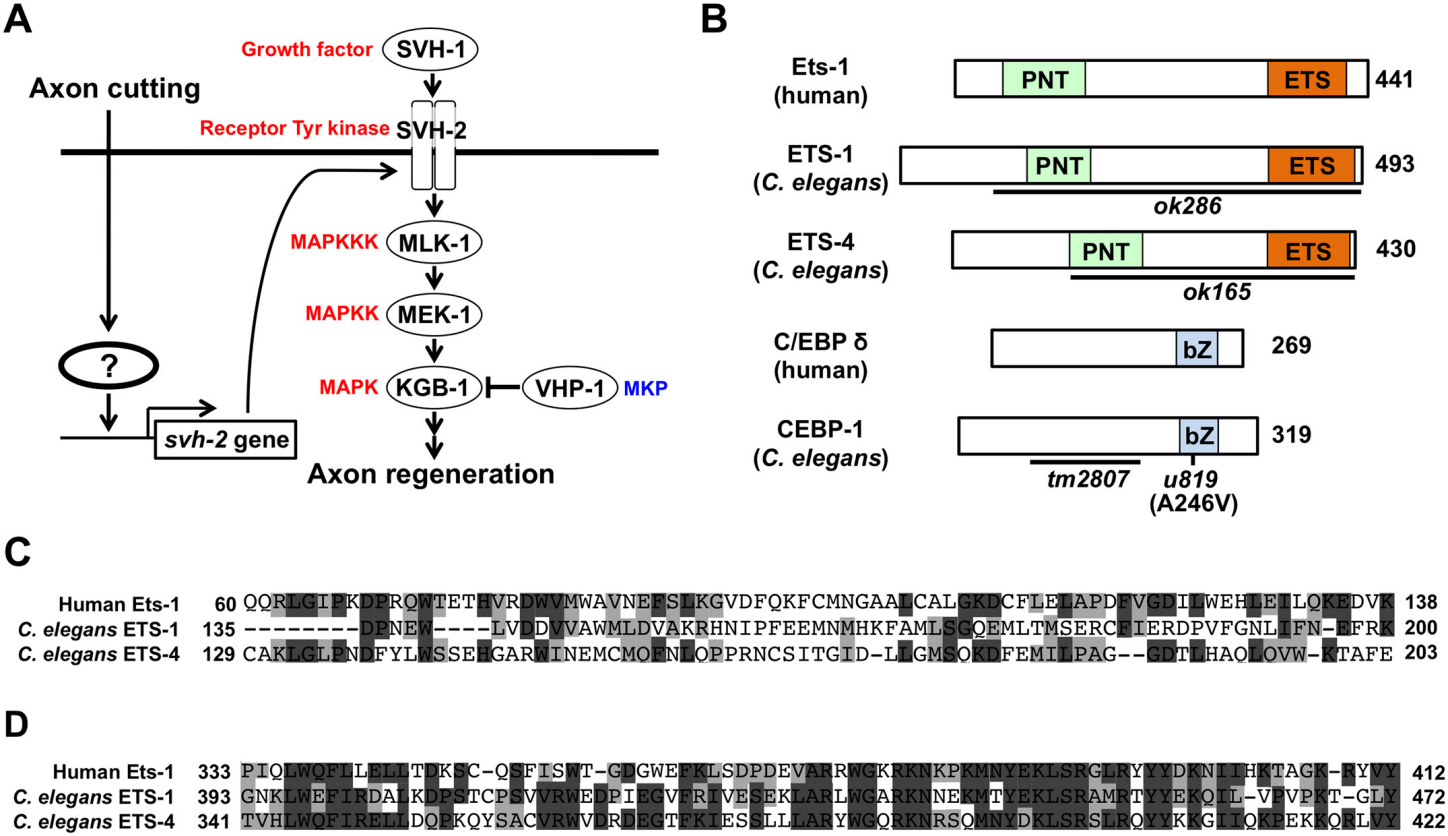
Identification of transcription factors acting in JNK cascade in *C*. *elegans*. (A) The JNK MAPK pathway is required for axon regeneration in *C*. *elegans*. Activation of SVH-2 receptor tyrosine kinase by SVH-1 growth factor activates the JNK pathway [11]. Axon cutting by laser microsurgery induces the transcription of the *svh-2* gene in severed neurons. However, the mechanism by which the *svh-2* gene is induced in response to axon cutting has been unknown. (B) Structures of ETS-1, ETS-4 and CEBP-1. Schematic diagrams of ETS-1, ETS-4, CEBP-1 and their human counterparts are shown. Domains are shown as follows: a pointed domain (PNT), an Ets DNA-binding domain (ETS), and a basic leucine-zipper domain (bZ). The bold lines underneath indicate the extent of the deleted region in each deletion mutant. The *u819* point mutation site is indicated. (C) Comparisons of PNT domains among human Ets-1, *C*. *elegans* ETS-1 and ETS-4. Identical and similar residues are highlighted with black and gray shading, respectively. (D) Comparisons of Ets domains among human Ets-1, *C*. *elegans* ETS-1 and ETS-4. Identical and similar residues are highlighted with black and gray shading, respectively.

The nematode *Caenorhabditis elegans* has recently emerged as a genetic model for studying the molecular control of axon regeneration [4,5]. Recent genetic studies have demonstrated that *C*. *elegans* axon regeneration is regulated by the p38 and JNK MAP kinase (MAPK) pathways, which consist of DLK-1 (MAPKKK)–MKK-4 (MAPKK)–PMK-3 (MAPK) and MLK-1(MAPKKK)–MEK-1(MAPKK)–KGB-1 (MAPK), respectively [6–8]. The p38 MAPK signaling pathway promotes axon regeneration by activating the MAP kinase-activated protein kinase MAK-2, which functions to stabilize the mRNA encoding the C/EBP family transcription factor CEBP-1 [7]. CEBP-1 in turn promotes axon regeneration, although the specific targets that mediate this response remain unknown. MAPK cascades can be inactivated by members of the MAPK phosphatase (MKP) family [9]. In *C*. *elegans*, the *vhp-1* gene encodes a MKP that negatively regulates both the DLK-1–MKK-4–PMK-3 and MLK-1–MEK-1–KGB-1 MAPK pathways [8,10]. *vhp-1* mutant animals are arrested during larval development, due to hyperactivation of the MAPK pathways. Furthermore, axon regeneration is enhanced in *vhp-1* mutants [8]. In a previous effort to identify additional components involved in MAPK-mediated signaling, we isolated a number of *svh* (suppressor of *vh* *p-1*) genes, which function as suppressors of *vhp-1* larval lethality [11]. Two of these, *svh-1* and *svh-2*, encode a growth factor and its cognate receptor tyrosine kinase, respectively. SVH-1–SVH-2 signaling mediates the activation of the JNK cascade following axonal injury, a molecular event essential for neuronal regeneration but not for neuronal development. This specific effect on axon regeneration is determined by *svh-2* gene expression, which is induced following axon injury in severed neurons (Fig 1A) [11].

In the present study, we investigated *ets-4* and *cebp-1*, two genes that emerged from our previous screen, and examined their potential roles in the regulation of axon regeneration. We demonstrate that ETS-4 and CEBP-1 act as transcriptional activators of *svh-2* expression in response to axon injury. ETS-4 and CEBP-1 function as downstream effectors of the cAMP and Ca^2+^–p38 MAPK pathways, respectively. Our results indicate that the cAMP and Ca^2+^–p38 MAPK pathways, induced in response to axon injury, converge through the formation of an ETS-4–CEBP-1 transcription factor complex to transactivate *svh-2* gene expression and SVH-2 receptor expression, which in turn activates the JNK pathway. Thus, the cAMP and Ca^2+^–p38 MAPK signaling pathways induce a transcription factor complex that ultimately up-regulates the JNK pathway and promotes axon regeneration.

## Results

### ETS-4 is required for efficient axon regeneration

**Fig 2:**
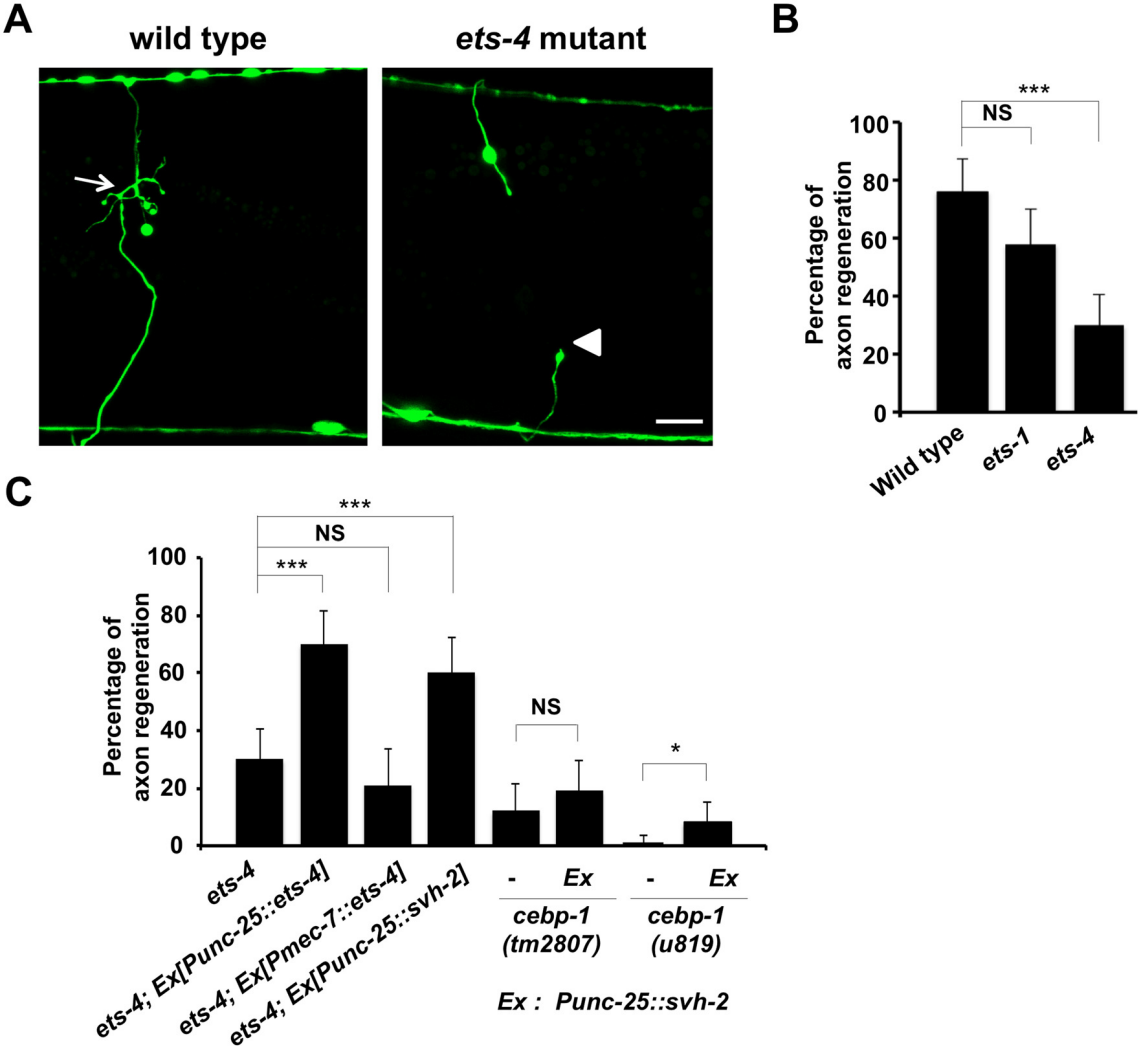
ETS-4 is required for efficient axon regeneration in *C*. *elegans*. (A) Representative D-type motor neurons in wild-type and *ets-4* mutant animals 24 hr after laser surgery. In wild-type animals, severed axons have regenerated growth cones (arrows). In *ets-4* mutants, the proximal end of axon failed to regenerate (arrowhead). Scale bar = 10 μm. (B, C) Percentages of axons that initiated regeneration 24 hr after laser surgery. Error bars indicate 95% SI. ***P<0.001; *P<0.05; NS, not significant.

To identify the transcription factors involved in axon injury-induced activation of *svh-2* expression, we asked if any of the *svh* genes encode transcription factors. Among our *svh* genes we identified *svh-5*, which encodes a member of the Ets transcription factor family and contains an Ets DNA binding domain and a PNT domain, a protein-protein interaction domain conserved in a subset of Ets proteins (Fig 1B, 1C and 1D) [12]. We therefore renamed *svh-5* as *ets-1*. To examine the effect of *ets-1* on axon regeneration, we assayed regrowth after laser axotomy in γ-aminobutyric acid (GABA)-releasing D-type motor neurons, which extend their axons from the ventral to the dorsal nerve cord (Fig 2A) [4,6]. In young adult wild-type animals, laser-severed axons were able to initiate regeneration within 24 hr (Fig 2A and 2B and S1 Table). Although the *ets-1(ok286)* deletion mutation (Fig 1B) slightly inhibited axon regeneration, this effect was not statistically significant (Fig 2B and S1 Table). The *C*. *elegans* genome contains ten *ets* genes, among which PNT domains are found in only ETS-1 and ETS-4 (Fig 1B and 1C) [13,14]. We found that in contrast to *ets-1*, the frequency of axon regeneration in *ets-4(ok165)* deletion mutants (Fig 1B) was reduced significantly (Fig 2A and 2B and S1 Table). The morphology of D-type motor neurons was normal in *ets-4* mutants. These results suggest that *ets-1* and *ets-4* are involved in *vhp-1*-mediated larval development and axon regeneration, respectively. To test whether ETS-4 can act in a cell-autonomous manner, we expressed the *ets-4* cDNA from the *unc-25* or *mec-7* promoters in *ets-4* mutants. The *ets-4* defect was rescued by expression of *ets-4* in D-type motor neurons by the *unc-25* promoter but not by expression in sensory neurons by the *mec-7* promoter (Fig 2C and S1 Table). These results demonstrate that ETS-4 functions cell autonomously in D-type motor neurons.

### ETS-4 and CEBP-1 are required for the transcriptional induction of the *svh-2* gene in response to axon injury

**Fig 3:**
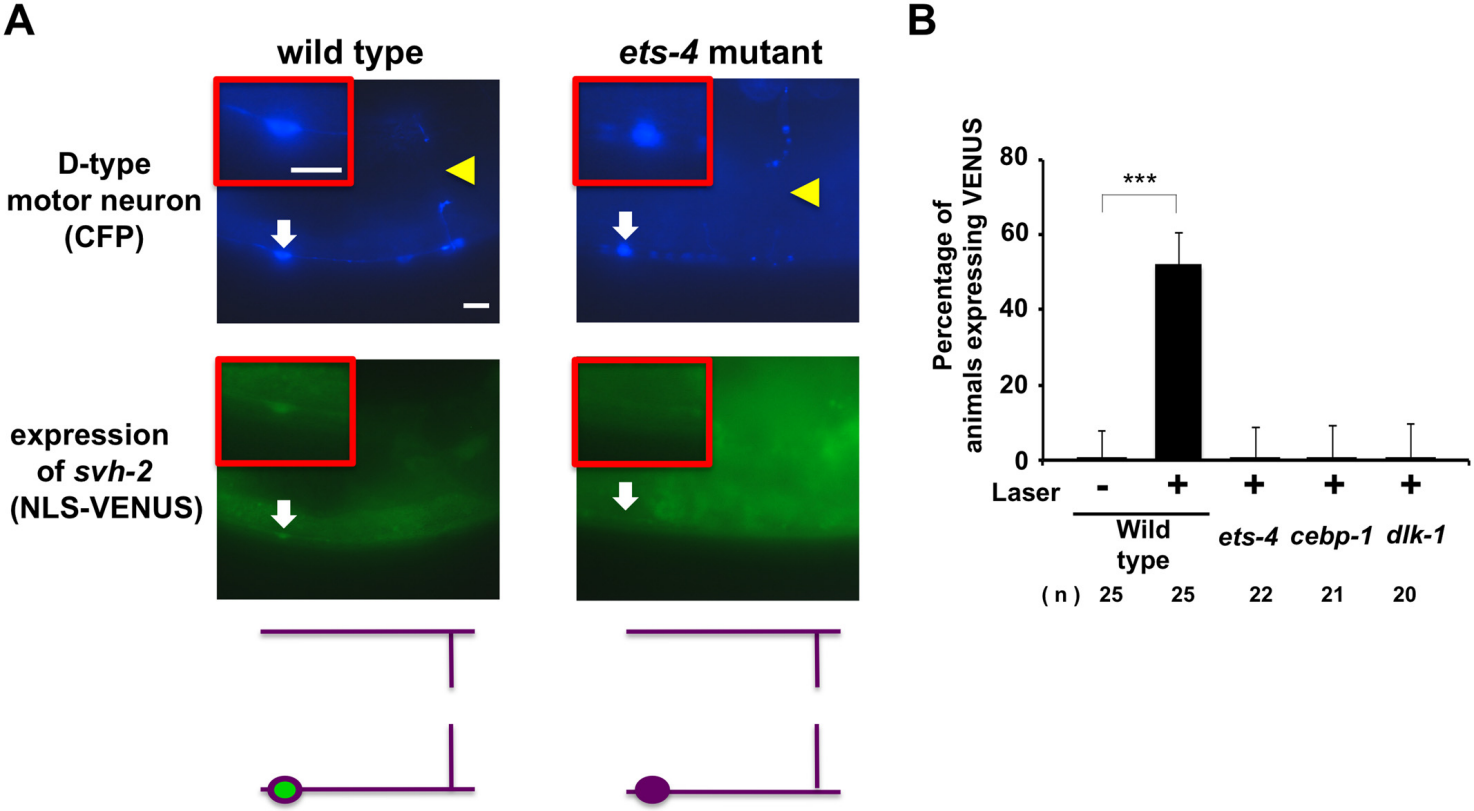
ETS-4 and CEBP-1 are required for the transcriptional induction of the *svh-2* gene in response to axon injury. (A) Induction of *Psvh-2*::*nls*::*venus* expression in D-type motor neurons by laser surgery. Expression of fluorescent proteins in D-type motor neurons of wild-type and *ets-4* mutants 3 hr after laser surgery are shown. Yellow arrowheads and white arrows indicate axon and cell bodies, respectively, of D-type neurons after laser surgery. D neurons are visualized by CFP under control of the *unc-25* promoter. Cell bodies of D-type neurons are magnified and shown within the upper boxes. A schematic representation of D-type motor neurons is shown below. Scale bars = 10 μm. (B) Percentages of animals expressing *svh-2*. Induction of *Psvh-2*::*nls*::*venus* expression in D-type motor neurons with (+) or without (-) laser surgery was assayed as described in Materials and Methods. The numbers (n) of animals examined are shown. Error bars indicate 95% SI. ***P<0.001.

Since expression of *svh-2* is induced by axon injury [11], we next examined whether ETS-4 is involved in *svh-2* expression in response to axon injury. For this purpose we used the transgene *Psvh-2*::*nls*::*venus*, which consists of the *svh-2* promoter driving the fluorescent protein VENUS fused to a nuclear localization signal (NLS) [11]. In wild-type animals, *Psvh-2*::*nls*::*venus* expression was induced in D-type neurons in 52% of the animals following laser surgery (Fig 3A and 3B and S1 Fig). In contrast, we found that the *ets-4(ok165)* mutation abolished *Psvh-2*::*nls*::*venus* induction in response to laser surgery in D-type neurons (Fig 3A and 3B). These results suggest that ETS-4 is a transcription factor required for axon injury-induced up-regulation of *svh-2* expression. If ETS-4 is required for axon regeneration through activation of *svh-2* gene expression, the *ets-4* defect should be suppressed by constitutive expression of *svh-2*. We replaced the *svh-2* promoter with the *unc-25* promoter to generate the *Punc-25*::*svh-2* transgene and introduced this as an extrachromosomal array into an *ets-4(ok165)* mutant. As expected, this construct was able to rescue the *ets-4(ok165)* animals (Fig 2C and S1 Table). These results support the idea that ETS-4 acts as a transcription factor regulating *svh-2* expression in response to axon injury.

Another gene obtained in our *svh* screen [11] was the *svh-8*/*cebp-1* gene, which encodes a homolog of mammalian C/EBP (CCAAT/enhancer-binding protein) (Fig 1B), and which indeed is known to be involved in axon regeneration [7]. We confirmed that animals having the *cebp-1(tm2807)* mutation (Fig 1B) are defective in axon regeneration in D-type motor neurons (Fig 2C and S1 Table). Therefore, we examined whether CEBP-1 is required for expression of the *svh-2* gene in response to axon injury. We found that in *cebp-1(tm2807)* mutants, laser surgery was unable to induce the expression of *Psvh-2*::*nls*::*venus* in D-type neurons (Fig 3B). Yan *et al*. showed that axotomy-induced signaling via the DLK-1–p38 MAPK pathway promoted the local translation and stabilization of *cebp-1* transcripts, accumulation of which is required for axon regeneration [7]. Since CEBP-1 functions downstream of the DLK-1–p38 MAPK pathway in axon regeneration, we investigated whether the DLK-1 pathway is involved in axon injury-induced expression of *svh-2*. We found that expression of the *svh-2* reporter in D-type neurons was not induced by axon injury in *dlk-1(km12)* null mutants (Fig 3B). These results support the possibility that the DLK-1–p38 MAPK pathway is involved in axon injury-induced expression of the *svh-2* gene. If the *svh-2* gene is the only transcriptional target of CEBP-1, the *cebp-1* defect should be suppressed by constitutive expression of *svh-2*. However, in contrast to *ets-4*, the *Punc-25*::*svh-2* transgene was unable to rescue the defect associated with the *cebp-1(tm2807)* mutation (Fig 2C and S1 Table). Partial rescue of the phenotype may be expected if CEBP-1 has additional transcriptional targets that act in parallel to promote regeneration. Consistent with this, the *Punc-25*::*svh-2* transgene weakly suppressed the regeneration defect caused by a different, stronger allele of *cebp-1(u819)* (Fig 2C and S1 Table). These results suggest that CEBP-1 has other target(s) in addition to *svh-2* that function in regeneration after axon injury.

### The *svh-2* promoter region is required for regeneration and gene expression in response to axon injury

**Fig 4:**
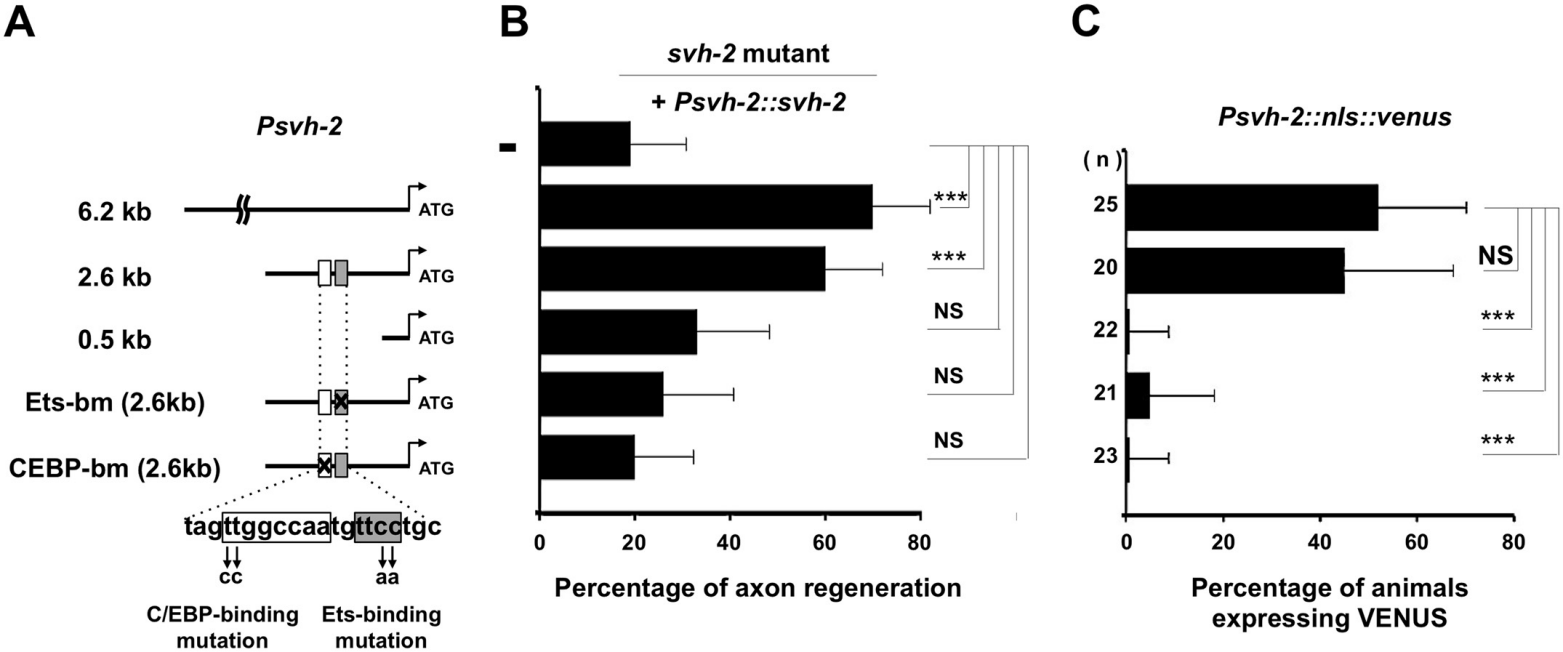
Promoter analysis of the *svh-2* gene. (A) The *svh-2* promoter region. Schematic diagram of the *svh-2* transgenes used in rescue experiments are shown. The positions of binding sites for Ets and C/EBP in the *svh-2* promoter are indicated by shaded and white boxes, respectively. (B) Identification of the *svh-2* promoter region required for axon regeneration. Each construct was transfected into *svh-2* mutants and analysed for axon regeneration. The percentages of axons that initiated regeneration 24 hr after laser surgery are shown. Error bars indicate 95% SI. ***P<0.001; NS, not significant. (C) Identification of the *svh-2* promoter region required for induction of *Psvh-2*::*nls*::*venus* expression in D-type motor neurons by laser surgery. Each construct was transfected into animals and analysed for *svh-2* gene induction in response to laser surgery. The percentages of animals expressing *Psvh-2*::*nls*::*venus* in D-type motor neurons following laser surgery are shown. The numbers (n) of animals examined are shown. Error bars indicate 95% SI. ***P<0.001; NS, not significant.

To determine the region in the *svh-2* promoter important for axon regeneration and gene induction following axon injury, we generated a series of *svh-2* promoter deletions (Fig 4A). We have previously demonstrated that a 6.2 kb region of the *svh-2* promoter, driving the *svh-2* gene, is sufficient to rescue defective axon regeneration in *svh-2* mutants (Fig 4B and S1 Table). We observed here that a 2.6 kb region of the promoter upstream of the translational start site is also sufficient to rescue this defect, but a 0.5 kb promoter region is not (Fig 4B and S1 Table). Consistent with this, we confirmed that expression of a *Psvh-2*::*nls*::*venus* construct in D-type neurons after exposure to axon injury could be induced by the 2.6 kb promoter region, but not by the 0.5 kb region (Fig 4C). These results indicate that the promoter region of the *svh-2* gene between 0.5 kb and 2.6 kb upstream of the translational start site is important for the transcriptional induction of the *svh-2* gene and for axon regeneration.

**Fig 5:**
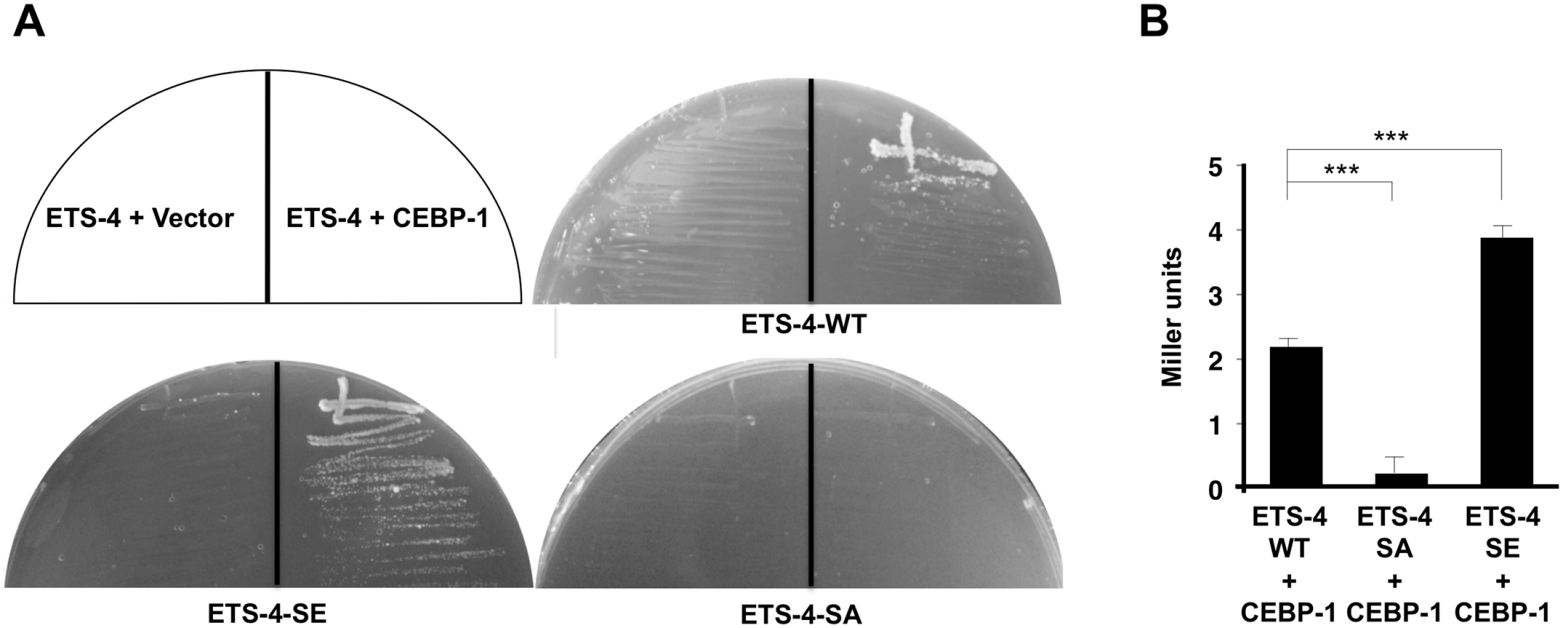
Interaction of ETS-4 with CEBP-1 by yeast two-hybrid assays. (A) The reporter yeast strain L40u was cotransformed with expression vectors encoding LexA DBD-ETS-4, LexA DBD-ETS-4(S73E), LexA DBD-ETS-4(S73A) and GAL4 AD-CEBP-1 as indicated. Yeasts carrying the indicated plasmids were grown on a selective plate lacking histidine and containing 80 mM 5-aminotriazole for 4 days. (B) The reporter yeast strain NMY51 was cotransformed with expression vectors as indicated. Yeasts carrying the indicated plasmids were grown and β-galactosidase activity was determined in cellular extracts as described in Materials and Methods. Backgrounds were determined by cotransforming cells with the control vector pACTII along with the corresponding pBTM116-ETS-4(WT, S73A or S73E,) and the β-galactosidase activities obtained were subtracted from each set of data. Values are given in Miller units. The data are combined from ten independent experiments and quantified by the Welch’s *t* test. ***P < 0.001. Error bars represent SEM.

To assess whether ETS-4 and CEBP-1 directly regulate *svh-2* expression, we searched the *svh-2* promoter region for Ets- and C/EBP-binding sites. Mammalian Ets binds the consensus sequence, 5’-GGAA/T-3’ [15], and the promoter region of the *svh-2* gene between 0.5 kb and 2.6 kb contains several possible Ets-binding motifs. This promoter region also has two C/EBP-binding motifs (5’-TTGNNCAA-3’) [16]. Of particular note, there is an Ets- and a C/EBP-binding site located in close proximity to one another at 1370 and 1378 base pairs upstream of the translational start site, respectively (Fig 4A). To determine whether these binding sites are required for axon regeneration and axon injury-induced expression of the *svh-2* gene, we converted the Ets consensus GGAA sequence to TTAA and the C/EBP consensus TTGGCCAA to CCGGCCAA (Fig 4A). We found that *svh-2* gene constructs carrying either of these point mutations in their promoter failed to rescue the *svh-2* defect in axon regeneration (Fig 4B and S1 Table). Furthermore, we found that axon injury-induced *Psvh-2*::*nls*::*venus* expression in D-type neurons was abolished by alteration of either of the Ets or C/EBP binding motif (Fig 4C). These results suggest that ETS-4 and CEBP-1 bind to the *svh-2* promoter via their respective binding site, and therein drive *svh-2* expression in response to axon injury. Thus, *svh-2* promoter activity appears to depend primarily on this combined Ets–C/EBP motif, raising the possibility that ETS-4 and CEBP-1 may physically interact at this site to drive *svh-2* gene expression. In a yeast two-hybrid assay we found that ETS-4 and CEBP-1 could indeed interact (Fig 5A and 5B), suggesting that ETS-4 may cooperate with CEBP-1 on the *svh-2* promoter to activate transcription.

### ETS-4 functions downstream of the cAMP pathway

**Fig 6:**
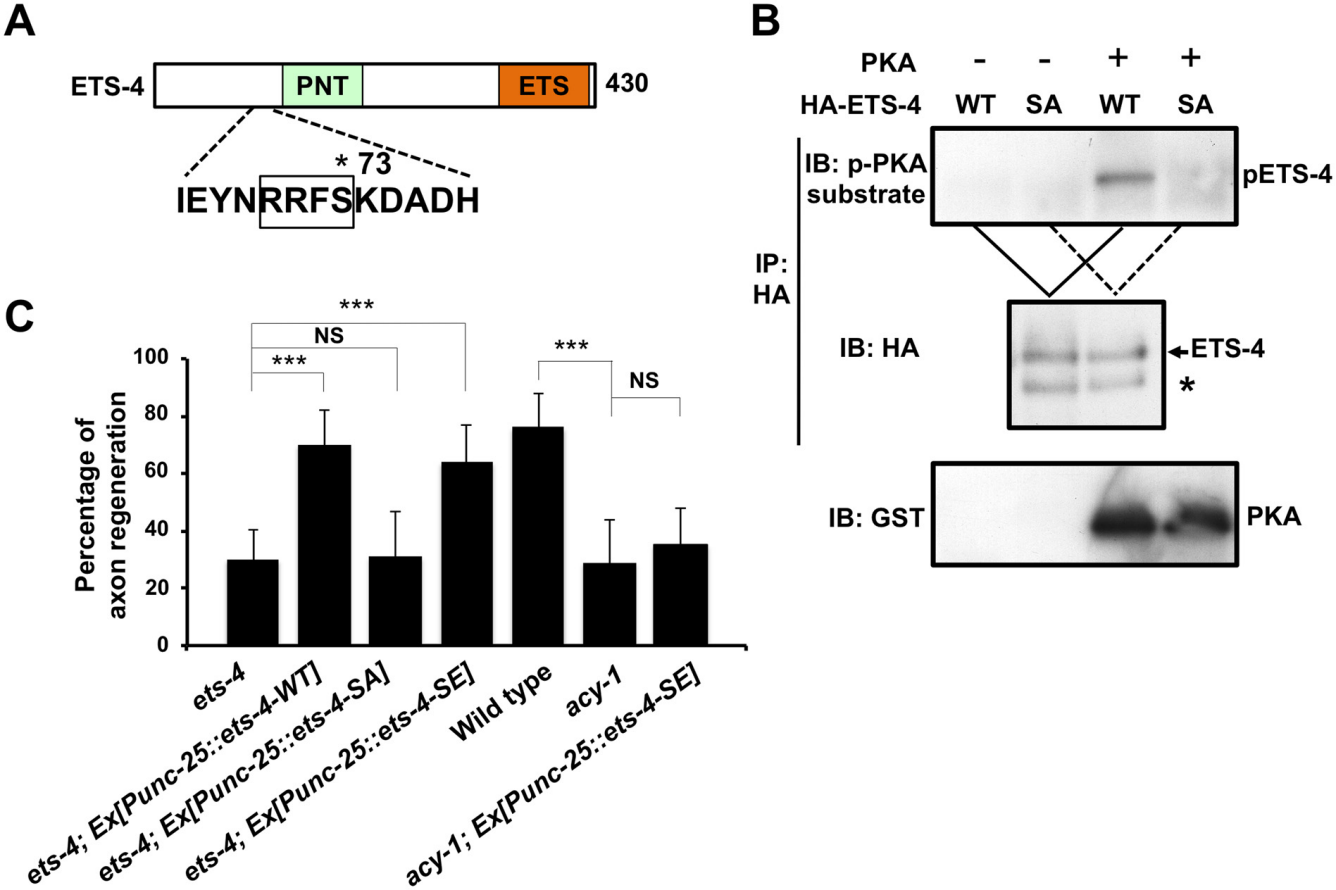
ETS-4 functions downstream of the cAMP pathway. (A) A schematic diagram of ETS-4. The amino acid sequence around a PKA phosphorylation consensus site is shown below. The consensus site for PKA phosphorylation is boxed. The Ser-73 residue is indicated by an asterisk. (B) PKA phosphorylates ETS-4 at Ser-73 in vitro. In vitro phosphorylation of ETS-4 protein by PKA is shown in the lower panels. COS-7 cells were transfected with HA-ETS-4 (WT) or HA-ETS-4(S73A) (SA), and cell lysates were immunoprecipitated with anti-HA antibody. The immunoprecipitates were divided and then subjected to in vitro kinase assays using recombinant GST-fused active PKA. Phosphorylated ETS-4 was detected by immunoblotting with anti-phospho-PKA substrate rabbit monoclonal antibody. Asterisk indicates a heavy chain of IgG. (C) Percentages of axons that initiated regeneration 24 hr after laser surgery. Error bars indicate 95% SI. ***P<0.001; NS, not significant.

How is ETS-4 regulated in axon regeneration? The functions of mammalian Ets transcription factors are regulated by phosphorylation [12,17]. As ETS-4 contains a protein kinase A (PKA) phosphorylation consensus sequence (Arg-Arg-Xxx-Ser) at Ser-73 near its PNT domain (Fig 6A), we asked whether PKA phosphorylates ETS-4 at this residue. We performed in vitro kinase assays with active PKA and immuno-purified HA-tagged ETS-4 and confirmed that PKA phosphorylated HA-ETS-4 (Fig 6B). To determine if PKA can phosphorylate ETS-4 on Ser-73, we generated a mutant form of ETS-4 [ETS-4(S73A)], in which Ser-73 is mutated to alanine. In vitro kinase assays showed that the S73A mutation abolished phosphorylation of ETS-4 by PKA (Fig 6B). These results demonstrate that PKA phosphorylates Ser-73 of ETS-4 in vitro.

We next addressed the biological importance of ETS-4 Ser-73 phosphorylation. When the phosphorylation-defective mutant ETS-4(S73A) was expressed under the control of the *unc-25* promoter in *ets-4(ok165)* null mutants, the defect in axon regeneration was not rescued (Fig 6C and S1 Table). In contrast, expression of a phospho-mimetic form of ETS-4, ETS-4(S73E), in *ets-4(ok165)* mutants by the *unc-25* promoter was able to rescue the regeneration defect (Fig 6C and S1 Table). Thus, phosphorylation of Ser-73 is important for the function of ETS-4 in the activation of the regeneration pathway.

We next asked how PKA-mediated phosphorylation might regulate ETS-4 in this regeneration pathway. Since Ser-73 is located near the PNT domain, which is a protein-protein interaction domain, we examined the effect of ETS-4 Ser-73 phosphorylation on its interaction with CEBP-1. We found that a non-phosphorylatable ETS-4(S73A) mutant form lost the ability to associate with CEBP-1, whereas a phosphorylation mimicking mutant ETS-4(S73E) was able to interact with CEBP-1 (Fig 5A and 5B). Furthermore, the interaction of ETS-4(S73E) with CEBP-1 was stronger than that of wild-type ETS-4 (Fig 5B). These results suggest that PKA-mediated phosphorylation of ETS-4 Ser-73 promotes the formation of an ETS-4–CEBP-1 complex.

**Fig 7:**
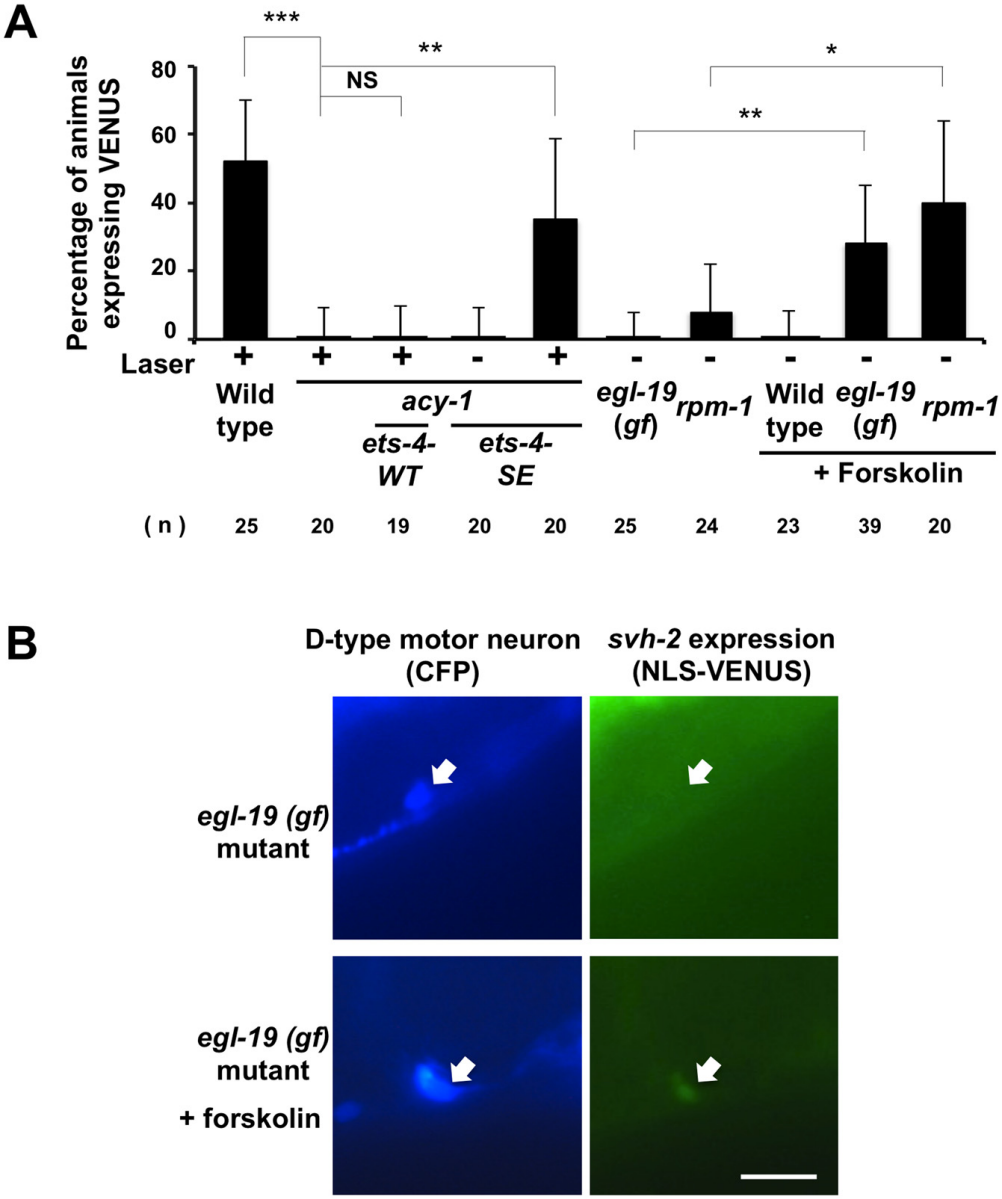
Activation of both cAMP and Ca^2+^–p38 MAPK signaling pathways is required for the induction of *svh-2* transcription. (A) Percentages of animals expressing *svh-2*. Induction of *Psvh-2*::*nls*::*venus* expression in D-type motor neurons with (+) or without (-) laser surgery was assayed as described in Materials and Methods. The numbers (n) of animals examined are shown. Error bars indicate 95% SI. ***P<0.001; **P<0.01; *P<0.05; NS, not significant. (B) *Psvh-2*::*nls*::*venus* expression in D-type motor neurons. Expression of fluorescent proteins in D-type motor neurons of *egl-19(gf)* mutants with or without forskolin treatment are shown. Arrows indicate cell bodies of D-type neurons. D neurons are visualized by CFP under control of the *unc-25* promoter. Scale bar = 10 μm.

PKA is activated by cAMP, and cAMP signals have been implicated in axonal regeneration in many systems [18–21]. The *C*. *elegans acy-1* gene encodes the neuronal adenylyl cyclase, and we observed that animals expressing a loss-of-function mutant, *acy-1(nu329)*, were defective in axon regeneration (Fig 6C and S1 Table) [18]. We also found that in animals carrying *acy-1(nu329)*, *Psvh-2*::*nls*::*venus* was not induced in D-type neurons in response to axon injury (Fig 7A). To examine whether ETS-4 functions downstream of cAMP in axon regeneration, we tested the effects of the phosphor-mimetic *ets-4* mutation on *acy-1* phenotypes. Expression of ETS-4(S73E) by the *unc-25* promoter failed to suppress the regeneration defect observed in *acy-1(nu329)* mutants (Fig 6C and S1 Table). This result is consistent with the fact that cAMP is known to be important for regeneration and regulates many pathways. Thus, ETS-4 is not the only target of cAMP signaling that functions in axon regeneration. In contrast to axon regeneration, we found that expression of ETS-4(S73E), but not wild-type ETS-4, was able to induce *svh-2* expression in *acy-1(nu329)* mutants (Fig 7A). However, this induction by ETS-4(S73E) was not constitutive and was not observed in the absence of axon injury. This result suggests that induction of *svh-2* expression requires the injury-dependent activation of both ETS-4 and CEBP-1 and that activation of ETS-4 alone is not sufficient to induce *svh-2* expression. Thus, ETS-4 is required for the induction of *svh-2* transcription in response to axonal injury, and this occurs downstream of cAMP signaling through PKA-mediated phosphorylation.

### Activation of both cAMP and Ca^2+^–p38 MAPK signaling pathways is required for the induction of *svh-2* transcription

Axotomy induces an intracellular increase in Ca^2+^ via the action of voltage-gated Ca^2+^ channels, and this can promote axon regeneration in a manner dependent on the DLK-1-p38 MAPK pathway [18,22]. Previous studies have shown that a gain-of-function (gf) mutation in a subunit of one voltage-gated Ca^2+^ channel, *egl-19(ad695gf)*, enhances Ca^2+^ influx in PLM neurons after axotomy and, subsequently, drives the formation of an active DLK-1 homomeric protein complex [22]. We therefore investigated whether activation of the p38-CEBP-1 pathway by the *egl-19(ad695gf)* mutation affects expression of the *svh-2* gene. We found that the *egl-19(ad695gf)* mutation did not cause constitutive expression of the *Psvh-2*::*nls*::*venus* reporter in D-type neurons (Fig 7A and 7B and S2 Fig). Since the ETS-4 transcription factor is regulated by the cAMP-PKA pathway, we examined the effect of activation of the cAMP pathway on the transcriptional induction of the *svh-2* gene. Treatment of animals with forskolin is expected to cause an increase in cAMP levels by activating adenylyl cyclase [18]. Forskolin treatment of wild-type animals not subjected to axon injury failed to induce *Psvh-2*::*nls*::*venus* expression in D-type neurons (Fig 7A). Thus, activation of either the p38-CEBP-1 or cAMP-ETS-4 pathway is not sufficient to induce transcription of the *svh-2* gene. We therefore speculated that simultaneous activation of both pathways might be required. Consistent with this, we found that when *egl-19(ad695gf)* mutants were treated with forskolin, 28% of the animals expressed the *Psvh-2*::*nls*::*venus* reporter in D-type neurons, even in the absence of axon injury (Figs 7A and 7B and S2).

We next examined whether the effect of the *egl-19* gf mutation on *svh-2* expression is mediated by the DLK-1 pathway. Activated DLK-1 kinase is targeted for degradation by the E3 ubiquitin ligase RPM-1, thereby modulating the duration of signaling [7]. Constitutive activation of the DLK-1 pathway induces developmental defects that mimic *rpm-1*mutants. Moreover, *rpm-1* mutants display a MAPK-dependent improvement in axon regeneration [8]. We found that the *rpm-1* mutation caused constitutive expression of the *svh-2* gene in D-type neurons when cultured in the presence of forskolin (Fig 7A). Taken together, these results suggest that induction of the *svh-2* gene is dependent on activation of both cAMP and Ca^2+^-DLK-1 signaling through an Ets-C/EBP transcription factor complex.

## Discussion

MAPK signaling cascades are evolutionally conserved in eukaryotes from yeast to mammals and play key roles in many aspects of neuronal development and function [23]. Recent genetic studies have shown that the DLK-1–MKK-4–PMK-3 p38 MAPK and the MLK-1–MEK-1–KGB-1 JNK pathways regulate axon regeneration in *C*. *elegans* [6–8]. The DLK MAPKKKs are required for axon regeneration in both *Drosophila melanogaster* and mice [24–26]. Similarly, JNK-mediated activation of c-Jun is important for axonal outgrowth of neurons in axotomized rat nodose and dorsal root ganglia (DRG) and mouse DRG [27,28]. These discoveries suggest that the core machinery that regulates axon regeneration is conserved from worms to mammals.

**Fig 8:**
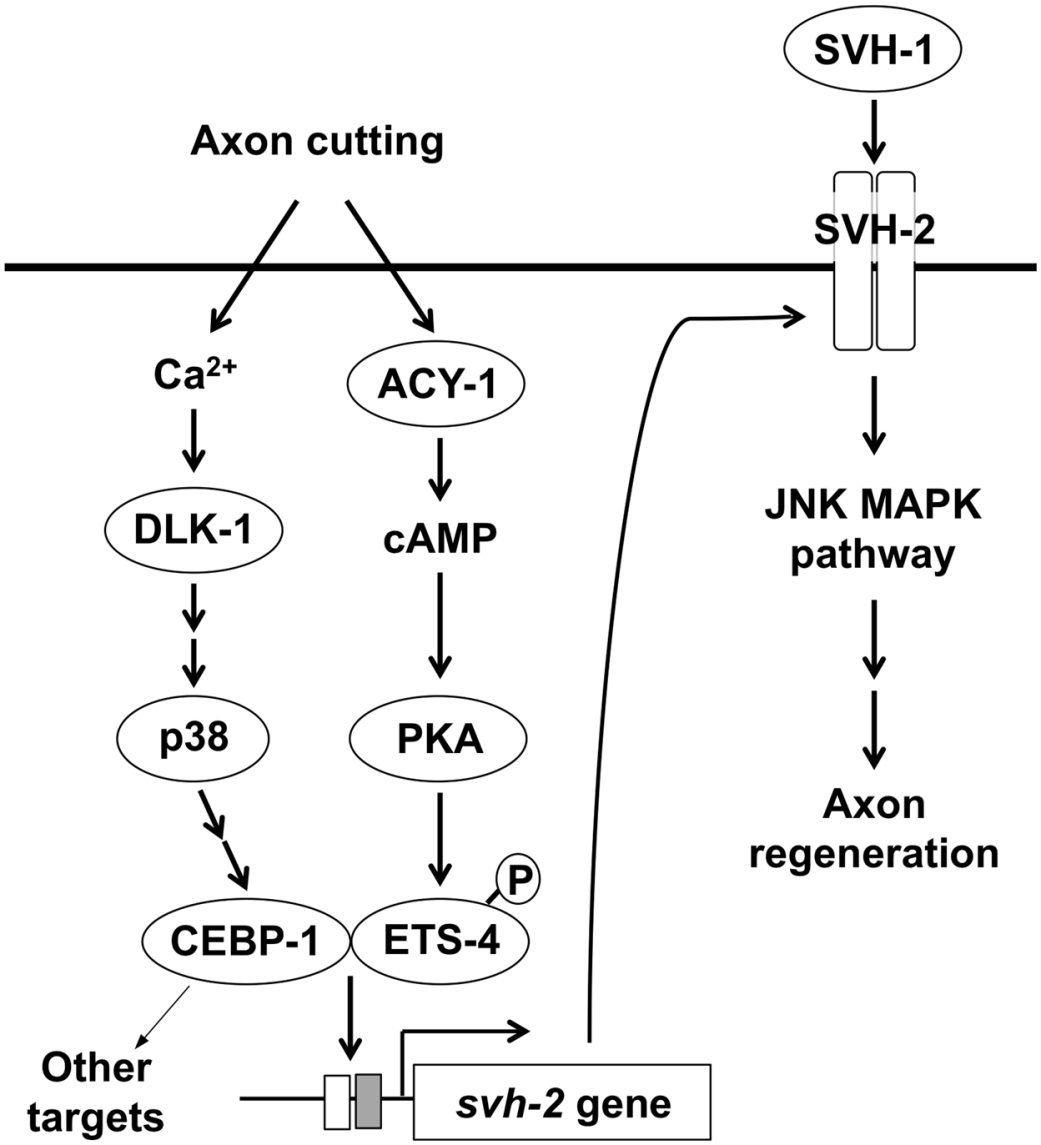
Schematic model for the regulation of JNK MAPK pathway by Ca^2+^–p38 MAPK and cAMP signaling pathways in axon regeneration.

In *C*. *elegans*, the DLK-1 p38 MAPK pathway promotes mRNA stability and local axonal translation of the bZip transcription factor CEBP-1 through the MAPKAP kinase MAK-2 [7]. Although the precise steps by which DLK-1 is activated in axon regeneration remain unknown, a Ca^2+^-dependent mechanism of activation has been recently described [22]. The JNK MAPK pathway is activated following axonal injury by growth factor-like SVH-1 engagement of its cognate receptor tyrosine kinase SVH-2 (Fig 8) [11]. SVH-1 belongs to the HGF/plasminogen family and SVH-2 is homologous to the HGF receptor Met, suggesting that SVH-1–SVH-2 functions as a ligand–receptor pair in axon regeneration. The *svh-1* gene is constitutively expressed in ADL sensory neurons in the head and SVH-1 acts on injured neurons. In contrast, expression of *svh-2* is induced by axonal injury [11]. SVH-1-SVH-2 signaling does not affect axon development per se, but rather is specific to axon regeneration. This specificity is determined by the injury-induced expression of the *svh-2* gene.

In this study, we found that the transcription factor CEBP-1 is required for the injury-induced transcriptional activation of *svh-2* expression. Although there remains the possibility that EGL-19–DLK-1– CEBP-1 pathway may activate *svh-2* expression through another MAPK, it is known that CEBP-1 acts in the p38 MAPK pathway [7]. Since SVH-2 functions in the JNK MAPK pathway [11], these results suggest the p38 MAPK pathway functions upstream of JNK MAPK activation in the response to axon injury (Fig 8). Enforced expression of *svh-2* in *cebp-1* mutants failed to efficiently suppress the defect in axon regeneration seen in these mutants, indicating that CEBP-1 transcriptionally regulates other targets in addition to the *svh-2* gene in response to p38 MAPK activation.

ETS-4 was also identified as another transcription factor involved in the activation of *svh-2* expression in response to axon injury. A previous study demonstrated that ETS-4 functions in lipid transport, lipid metabolism and innate immunity [14]. ETS-4 is a transcriptional regulator of aging, and shares transcriptional targets with the GATA and FOXO transcriptional regulators. An expression profiling study identified seventy ETS-4-target genes, 54 of which have identifiable Ets-binding consensus motifs in their promoter regions [14]. However, this analysis did not identify the *svh-2* gene as an ETS-4 target, presumably because ETS-4-dependent *svh-2* expression occurs only in severed neurons and not under normal conditions. We show that ETS-4 acts downstream of cAMP signaling, and that transcriptional up-regulation of *svh-2* by ETS-4 requires PKA-dependent phosphorylation at Ser-73. However, a mutant of ETS-4 that mimics constitutive phosphorylation of the Ser-73 residue failed to rescue the defect in axon regeneration in *acy-1* mutants defective in cAMP production. These results suggest that cAMP activates other pathways, in addition to the SVH-2–JNK MAPK pathway, that are required for axon regeneration.

We demonstrate that phosphorylation of ETS-4 by PKA leads to the formation of a complex containing ETS-4 and CEBP-1, and that this protein-protein interaction is necessary for activation of *svh-2* expression in response to axon injury. It has been shown for mammals that direct interactions between C/EBPs and specific Ets family members are important for eosinophil lineage determination [29]. In this case, the physical interaction between Ets-1 and C/EBPα proteins is mediated by their DNA-binding domains, and these complexes transactivate their target genes by high-affinity binding to combined motifs in the target promoters. Similarly, the *svh-2* promoter region has an Ets- and a C/EBP-binding site located in close proximity to one another and these binding sites are required for axon injury-induced expression of the *svh-2* gene. It is tempting to speculate that the high affinity of the CEBP-1–ETS-4 interaction is important for synergy between the two factors. We conclude that CEBP-1 is a biologically important and relevant interacting partner of ETS-4 that is involved in the transcriptional activation of *svh-2* gene expression.

Similar to vertebrate neurons, increased Ca^2+^ and cAMP facilitate axon regeneration in severed *C*. *elegans* neurons [18]. It is likely that some effects of elevated Ca^2+^ or cAMP on axon regeneration are mediated at the transcriptional level [30]. Interestingly, CEBP-1 acts downstream of the Ca^2+^–p38 MAPK pathway and ETS-4 functions downstream of the cAMP signaling pathway. Based on our findings, we propose a model wherein axon injury initiates cAMP signaling and the Ca^2+^–p38 MAPK pathway, which together function to induce the formation of an ETS-4–CEBP-1 transcription factor complex. This complex binds to the *svh-2* promoter to induce *svh-2* expression, and SVH-1 signaling via SVH-2 then activates the JNK pathway (Fig 8). Thus, two injury-signaling pathways (p38 MAPK and cAMP/PKA) converge to regulate expression of the *svh-2* gene after injury, which ultimately promotes axon regeneration. The identification and characterization of the signaling pathways that regulate axon regeneration in *C*. *elegans* should yield numerous insights into the mechanisms used by nervous systems to regulate similar processes across metazoans.

## Materials and Methods

### *C*. *elegans* strains

*C*. *elegans* strains used in this study are listed in S2 Table. All strains were maintained on nematode growth medium (NGM) plates and fed with bacteria of the OP50 strain, as described previously [31].

### Axotomy

Axotomy and microscopy were performed as described previously [11]. All animals were subjected to axotomy at the young adult stage. The imaged commissures that had growth cones or small branches present on the proximal fragment were counted as “regenerated”. The proximal fragments that showed no change after 24 hr were counted as “no regeneration”. A minimum of 20 individuals with 1–3 axotomized commissures were observed for most experiments.

### Microscopy

Standard fluorescent images of transgenic worms were observed under a Zeiss Plan-APOCHROMAT 63 X objective of a Zeiss Axioplan II fluorescent microscope and photographed with a Hamamatsu 3CCD camera. Confocal fluorescent images were taken on an Olympus FV500 confocal laser scanning microscope with a 40 X objective.

### Plasmids

*Punc-25*::*ets-4* and *Pmec-7*::*ets-4* were generated by inserting the *ets-4* cDNA isolated from a cDNA library into the pCZ325 vector and pPD52.102 vectors, respectively. *Punc-25*::*ets-4(S73A)* and *Punc-25*::*ets-4(S73E)* were made by oligonucleotide-directed PCR using *Punc-25*::*ets-4* as a template and the mutations were verified by DNA sequencing. pLexA-ETS-4, pLexA-ETS-4(S73A) and pLexA-ETS-4(S73E) plasmids were constructed by inserting the wild-type and mutagenized *ets-4* cDNAs into the pBTM116 vector. The *Psvh-2(6*.*2 kb)*::*nls*::*venus* plasmid has been described previously [11]. For the construction of *Psvh-2(2*.*6 kb)*::*nls*::*venus* and *Psvh-2(0*.*5 kb)*::*nls*::*venus* plasmids, *Psvh-2(6*.*2 kb)*::*nls*::*venus* was digested with either *Hind*III or *Psi*I, respectively, and then self-ligated. *Psvh-2(Ets-bm)*::*nls*::*venus* and *Psvh-2(C/EBP-bm)*::*nls*::*venus* plasmids were made by oligonucleotide-directed PCR using *Psvh-2(2*.*6 kb)*::*nls*::*venus* as a template and the mutations were verified by DNA sequencing. These mutated promoters were then used to make *Psvh-2(6*.*2 kb)*::*svh-2*, *Psvh-2(2*.*6 kb)*::*svh-2*, *Psvh-2(0*.*5 kb)*::*svh-2*, *Psvh-2(Ets-bm)*::*svh-2* and *Psvh-2(C/EBP-bm)*::*svh-2*, respectively. The pCMV-HA-ETS-4 plasmid was made by inserting the *ets-4* cDNA into the pCMV-HA vector. The pACTII-CEBP-1 plasmid was made by inserting the *cebp-1* cDNA, isolated from a *C*. *elegans* cDNA library by PCR, into the pACTII vector. The *lin-15* plasmid is a gift from Dr. S. Takagi (Nagoya University). Other plasmids, including *Pttx-3*::*gfp*, *Pmyo-2*::*dsredm*, *Punc-25*::*cfp* and *Punc-25*::*svh-2* have been described previously [11, 32].

### Transgenic animals

Transgenic animals were obtained as described [33]. *Psvh-2(6*.*2 kb)*::*svh-2* (25 ng/μl), *Psvh-2(2*.*6 kb)*::*svh-2* (25 ng/μl), *Psvh-2(0*.*5 kb)*::*svh-2* (25 ng/μl), *Psvh-2(Ets-bm)*::*svh-2* (25 ng/μl) and *Psvh-2(C/EBP-bm)*::*svh-2* (25 ng/μl) plasmids were used in kmEx529 [*Psvh-2(6*.*2 kb)*::*svh-2* + *Pmyo-2*::*dsredm*], kmEx530 [*Psvh-2(2*.*6 kb)*::*svh-2* + *Pmyo-2*::*dsredm*], kmEx531 [*Psvh-2(0*.*5 kb)*::*svh-2* + *Pmyo-2*::*dsredm*], kmEx532 [*Psvh-2(Ets-bm)*::*svh-2* + *Pmyo-2*::*dsredm*], kmEx533 [*Psvh-2(C/EBP-bm)*::*svh-2 + Pmyo-2*::*dsredm*], respectively. *Punc-25*::*ets-4* (25 ng/μl), *Pmec-7*::*ets-4* (25 ng/μl), *Punc-25*::*ets-4(S73A)* (25 ng/μl) and *Punc-25*::*ets-4(S73E)* (25 ng/μl) plasmids were used in kmEx534 [*Punc-25*::*ets-4 + Pmyo-2*::*dsredm*], kmEx535 [*Pmec-7*::*ets-4 + Pmyo-2*::*dsredm*], kmEx536 [*Punc-25*::*ets-4(S73A) + Pmyo-2*::*dsredm*], kmEx537 [*Punc-25*::*ets-4(S73E) + Pmyo-2*::*dsredm*], kmEx544 [*Punc-25*::*ets-4 + Pttx-3*::*gfp*] and kmEx545 [*Punc-25*::*ets-4(S73E) + Pttx-3*::*gfp*], respectively. *Punc-25*::*cfp* (50 ng/μl), *Psvh-2(6*.*2 kb)*::*nls*::*venus* (75 ng/μl), *Psvh-2(2*.*6 kb)*::*nls*::*venus* (75 ng/μl), *Psvh-2(Ets-bm)*::*nls*::*venus* (75 ng/μl) and *Psvh-2(C/EBP-bm)*::*nls*::*venus* (75 ng/μl) plasmids were used in kmEx538 (*Punc-25*::*cfp* + *lin-15*), kmEx539 [*Psvh-2(6*.*2 kb)*::*nls*::*venus* + *Pmyo-2*::*dsredm*], kmEx540 [*Psvh-2(2*.*6 kb)*::*nls*::*venus* + *Pmyo-2*::*dsredm*], kmEx541 [*Psvh-2(0*.*5 kb)*::*nls*::*venus* + *Pmyo-2*::*dsredm*], kmEx542 [*Psvh-2(Ets-bm)*::*nls*::*venus* + *Pmyo-2*::*dsredm*] and kmEx543 [*Psvh-2(C/EBP-bm)*::*nls*::*venus* + *Pmyo-2*::*dsredm*], respectively.

### Yeast two-hybrid assays

pBTM116-ETS-4(WT, S73E, S73A) and pACTII-CEBP-1 plasmids were cotransformed into the *Saccharomyces cerevisiae* reporter strain L40u [*MATa trp1 ura3 leu2 his3 LYS2*::*(lexAop)*_*4*_ *-HIS3*] and allowed to grow on SC-Leu-Trp plates. Transformants grown on these plates were streaked out onto SC-Leu-Trp-His plates containing 80 mM 5-aminotriazole and incubated at 30℃ for 4 days. For β-galactosidase assays, the NMY51 [*MATa trp1 ura3*::*(lexAop)*_*8*_ *-lacZ leu2 his3 LYS2*::*(lexAop)*_*4*_ *-HIS3 ade2*::*(lexAop)*_*8*_ *-ADE2 GAL4*] strain (Dualsystems Biotech) was used as the host strain. The β-galactosidase assay was performed as described previously [34].

### DAPI staining

To examine nuclei of D neuron cells, we fixed worms in 4% paraformaldehyde for 1 hr and permeabilized with methanol for 5 min. Next, the worms were stained with 0.1 μg/ml of the DNA-binding dye 4,6-diamidino-2-phenyl-indole and mounted on 2% agarose slides for viewing using fluorescence imaging.

### Quantification of VENUS expression

Expression of VENUS fluorescence was quantified using the ImageJ program (NIH). The cell bodies of severed D neurons were outlined with closed polygons and the fluorescent intensities within these areas were determined (I_s_). The cell bodies of unsevered D neurons in the same animal were analyzed similarly as controls (I_u_). To determine the background intensity of each cell, the same polygon was placed in the area neighboring the cell body and fluorescence measured (I_bs_ and I_bu_, respectively). The relative signal intensity (I_r_) was calculated as (I_s_-I_bs_)/(I_u_-I_bu_). Cells having an I_r_>5 were categorized as “expressed”.

### Biochemical analyses

Transfection of transgenes into COS-7 cells, preparation of the cell lysates and immunoblotting procedures have been described previously [10]. Anti-phospho-PKA substrate (RRX(p)S/(p)T) [(p), phosphorylated] rabbit monoclonal antibody (100G7E), was purchased from Cell Signaling Technology.

### Statistical analysis

Statistical analyses were carried out as described previously [11]. Briefly, confidence intervals (95%) were calculated by the modified Wald method and two-tailed P values were calculated using Fisher’s exact test (http://www.graphpad.com/quickcalcs/contingency1/). The Welch’s *t* -test was performed by using *t*-test calculator (http://www.graphpad.com/quickcalcs/ttest1/).

## Supporting Information

S1 Fig Induction of *Psvh-2*::*nls*::*venus* expression in D-type motor neurons by laser surgery. Expression of fluorescent proteins in D-type motor neurons of wild-type animals 3 hr after laser surgery are shown. Yellow arrowheads and white arrows indicate axon and cell bodies, respectively, of D-type neurons after laser surgery. D neurons are visualized by CFP under control of the *unc-25* promoter. Nuclei are visualized by DAPI staining. Cell bodies of D-type neurons are magnified and shown in the lower panels. A schematic representation of D-type motor neurons is shown in the right. Scale bars = 10 μm.
(TIF)

S2 Fig *Psvh-2*::*nls*::*venus* expression in *egl-19(gf)* mutants. Expression of fluorescent proteins in D-type motor neurons of *egl-19(gf)* mutants with or without forskolin treatment are shown. Arrows indicate cell bodies of D-type neurons. D neurons are visualized by CFP under control of the *unc-25* promoter. Nuclei are visualized by DAPI staining. Scale bar = 10 μm.
(TIF)

S1 Table Raw data for genotypes tested by axotomy. (PDF)

S2 Table Strains used in this study. (PDF)
