# Supplementary figures and images for: Axon Regeneration Is Regulated by Ets–C/EBP Transcription Complexes Generated by Activation of the cAMP/Ca2+ Signaling Pathways
Source: PLoS Genet. 2015 Oct 20;11(10):e1005603. doi: 10.1371/journal.pgen.1005603 (PMC4618690; doi:10.1371/journal.pgen.1005603)

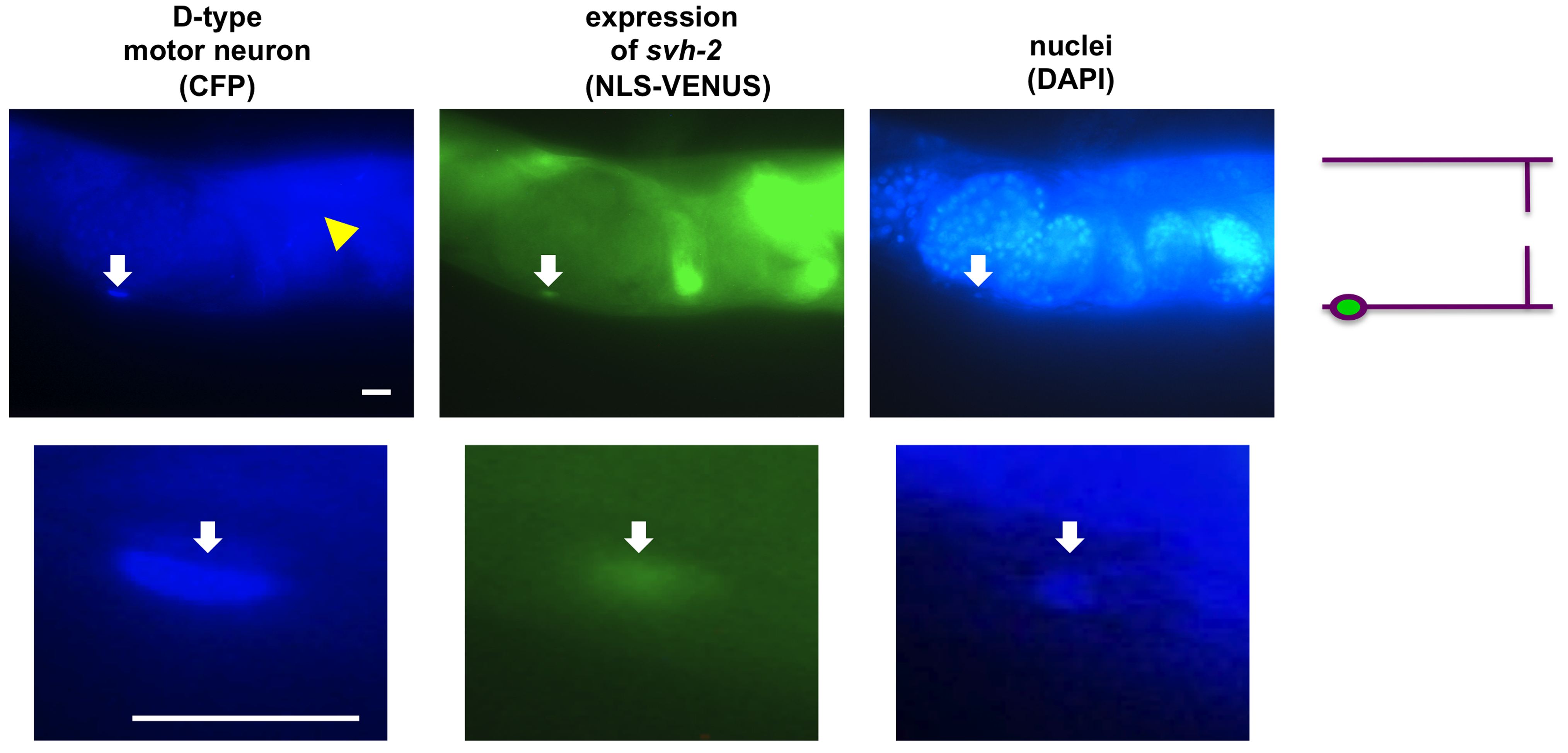

Supplement: S1 Fig — Expression of fluorescent proteins in D-type motor neurons of wild-type animals 3 hr after laser surgery are shown. Yellow arrowheads and white arrows indicate axon and cell bodies, respectively, of D-type neurons after laser surgery. D neurons are visualized by CFP under control of the unc-25 promoter. Nuclei are visualized by DAPI staining. Cell bodies of D-type neurons are magnified and shown in the lower panels. A schematic representation of D-type motor neurons is shown in the right. Scale bars = 10 μm. (TIF) [file pgen.1005603.s001.tif]

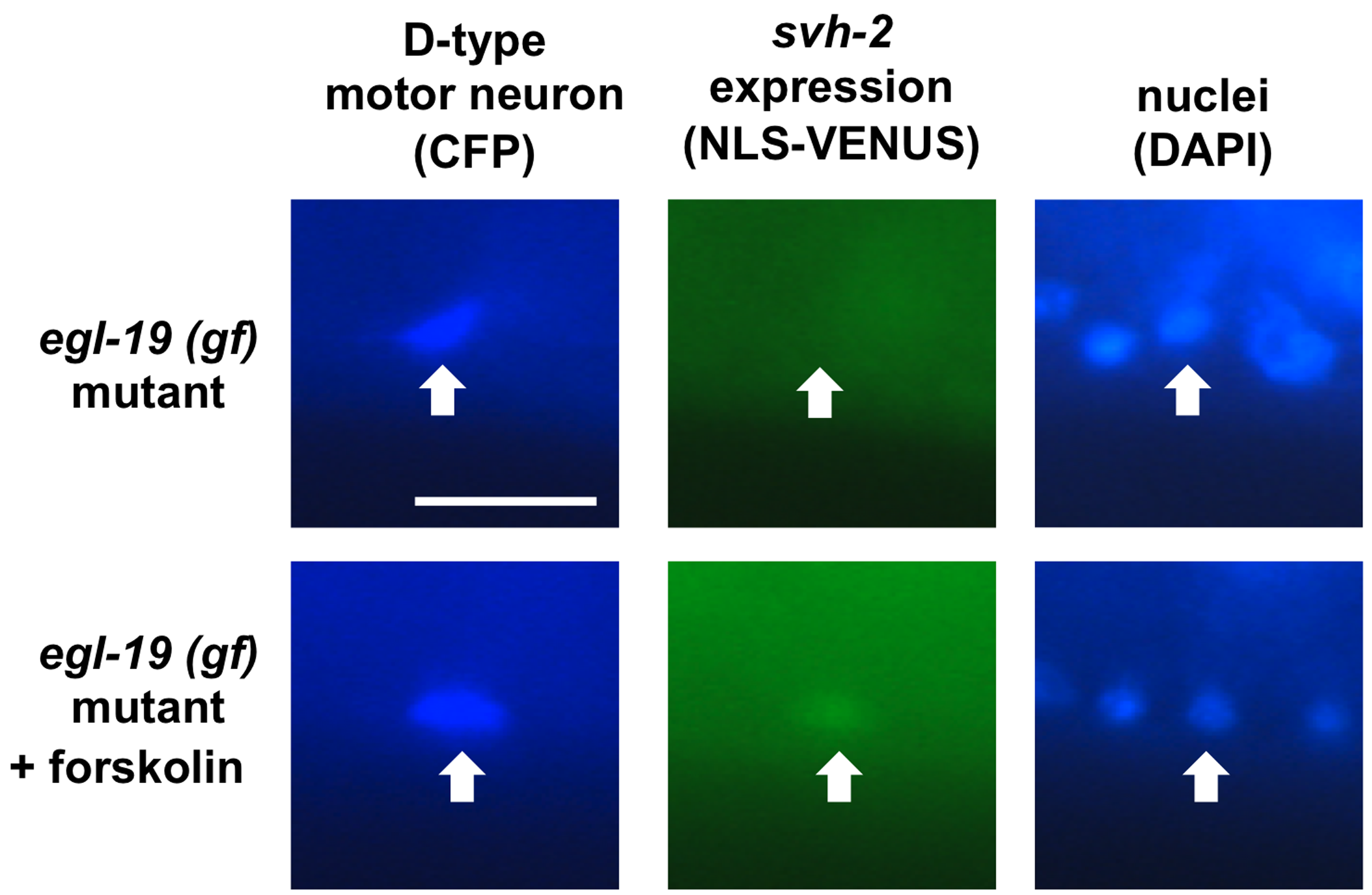

Supplement: S2 Fig — Expression of fluorescent proteins in D-type motor neurons of egl-19(gf) mutants with or without forskolin treatment are shown. Arrows indicate cell bodies of D-type neurons. D neurons are visualized by CFP under control of the unc-25 promoter. Nuclei are visualized by DAPI staining. Scale bar = 10 μm. (TIF) [file pgen.1005603.s002.tif]
