# Supplementary material for: Axon Regeneration Is Regulated by Ets–C/EBP Transcription Complexes Generated by Activation of the cAMP/Ca2+ Signaling Pathways
Source: PLoS Genet. 2015 Oct 20;11(10):e1005603. doi: 10.1371/journal.pgen.1005603 (PMC4618690; doi:10.1371/journal.pgen.1005603)
Supplement: S1 Table — (PDF) [file pgen.1005603.s003.pdf]

S1 Table. Raw data for genotypes tested by axotomy

Strain	Genotype	No. of animals	No. of axons	No. of regeneration	P vs. control
KU501	*juIs76*	27	54	41 (76%)	-
KU544	*ets-1; juIs76*	30	60	35 (58%)	0.0503[a]
KU545	*ets-4; juIs76*	63	115	34 (30%)	< 0.0001[a]
KU546	*ets-4; juIs76; Punc-25::ets-4*	27	56	39 (70%)	< 0.0001[b]
KU547	*ets-4; juIs76; Pmec-7::ets-4*	26	52	11 (21%)	0.3464[b]
KU548	*ets-4; juIs76; Punc-25::svh-2*	33	55	33 (60%)	0.0002[b]
KU549	*ets-4; juIs76; Punc-25::ets-4(S73A)*	28	51	16 (31%)	0.8554[b]
KU550	*ets-4; juIs76; Punc-25::ets-4(S73E)*	28	50	32 (64%)	< 0.0001[b]
KU551	*cebp-1(tm2807); juIs76*	28	59	7 (12%)	< 0.0001[a]
KU552	*cebp-1(tm2807); juIs76; Punc-25::svh-2*	34	73	14 (19%)	0.3397[c]
KU553	*acy-1; juIs76*	31	55	16 (29%)	< 0.0001[a]
KU503	*svh-2; juIs76*	26	59	11 (19%)	< 0.0001[a]
KU555	*svh-2; juIs76; Psvh-2(6.2 kb)::svh-2*	27	53	37 (70%)	< 0.0001[d]
KU556	*svh-2; juIs76; Psvh-2(2.6 kb)::svh-2*	31	60	36 (60%)	< 0.0001[d]
KU557	*svh-2; juIs76; Psvh-2(0.5 kb)::svh-2*	32	55	18 (33%)	0.0912[d]
KU558	*svh-2; juIs76; Psvh-2(Ets-bm)::svh-2*	32	50	13 (26%)	0.3661[d]
KU559	*svh-2; juIs76; Psvh-2(CEBP-bm)::svh-2*	38	56	11 (20%)	1[d]
KU570	*cebp-1(u819); juIs76*	31	62	0 (0%)	< 0.0001[a]
KU571	*cebp-1(u819); juIs76; Punc-25::svh-2*	38	76	6 (8%)	0.033[e]
KU572	*acy-1; juIs76; Punc-25::ets-4(S73E)*	47	84	29 (35%)	0.5798[f]

a: KU501 as a control, b: KU545 as a control, c: KU551 as a control, d: KU503 as a control, e: KU570 as a control, f: KU553 as a control.
