# Supplementary material for: Axon Regeneration Is Regulated by Ets–C/EBP Transcription Complexes Generated by Activation of the cAMP/Ca2+ Signaling Pathways
Source: PLoS Genet. 2015 Oct 20;11(10):e1005603. doi: 10.1371/journal.pgen.1005603 (PMC4618690; doi:10.1371/journal.pgen.1005603)
Supplement: S2 Table — (PDF) [file pgen.1005603.s004.pdf]

S2 Table. Strains used in this study

Strain	Genotype
KU501	*juIs76* II
KU503	*juIs76* II; *svh-2(tm737)* X
KU544	*juIs76* II; *ets-1(ok286)* X
KU545	*juIs76* II; *ets-4(ok165)* X
KU551	*juIs76* II; *cebp-1(tm2807)* X
KU553	*juIs76* II; *acy-1(nu329)* III
KU555	*juIs76* II; *svh-2(tm737)* X; *kmEx529*
KU556	*juIs76* II; *svh-2(tm737)* X; *kmEx530*
KU557	*juIs76* II; *svh-2(tm737)* X; *kmEx531*
KU558	*juIs76* II; *svh-2(tm737)* X; *kmEx532*
KU559	*juIs76* II; *svh-2(tm737)* X; *kmEx533*
KU546	*juIs76* II; *ets-4(ok165)* X; *kmEx534*
KU547	*juIs76* II; *ets-4(ok165)* X; *kmEx535*
KU548	*juIs76* II; *ets-4(ok165)* X; *kmEx501*
KU552	*juIs76* II; *cebp-1(tm2807)* X; *kmEx501*
KU549	*juIs76* II; *ets-4(ok165)* X; *kmEx536*
KU550	*juIs76* II; *ets-4(ok165)* X; *kmEx537*
KU560	*lin-15 (n765)* X; *kmEx538*; *kmEx539*
KU561	*lin-15 (n765)* X; *kmEx538*; *kmEx540*
KU562	*lin-15 (n765)* X; *kmEx538*; *kmEx541*
KU563	*lin-15 (n765)* X; *kmEx538*; *kmEx542*
KU564	*lin-15 (n765)* X; *kmEx538*; *kmEx543*
KU565	*ets-4(ok165) lin-15 (n765)* X*; kmEx538*
KU566	*cebp-1(tm2807) lin-15* (*n765*) X; *kmEx538*
KU567	*dlk-1(km12)* I; *lin-15 (n765)* X; *kmEx538*
KU568	*acy-1(nu329)* III; *lin-15 (n765)* X; *kmEx538*
KU569	*egl-19(ad695)* IV; *lin-15 (n765)* X; *kmEx538*
